# Does polycystic ovary syndrome put women at higher risk of developing additional medical conditions?

**Published:** 2022-07

**JUNE 08, 2022 -** Polycystic ovary syndrome (PCOS) is the most common endocrine disorder in women of reproductive age. In a study published in *Acta Obstetricia et Gynecologica Scandinavica*, women with PCOS were more likely than other women to also be diagnosed with migraine, hypertension, tendinitis, osteoarthritis, and endometriosis. Affected women were also using medications more often and reported their own health to be poorer than women without PCOS.

The study included 246 women with PCOS symptoms or diagnoses and 1,573 controls who were surveyed during their late reproductive years at age 46.

“PCOS is often labelled as a reproductive concern; however, in most cases this is well managed with fertility treatments. Our study underscores the need for health professionals to acknowledge the risk for several comorbidities and increased health burden related to this common syndrome,” said senior author professor Terhi T. Piltonen, MD, PhD, of the University of Oulu, in Finland. “Women should also be aware of this risk, and they should be supported by early diagnosis and treatment.”

*Full Citation: “Women with polycystic ovary syndrome are burdened with multimorbidity and medication use independent of body mass index at late fertile age: a population-based cohort study” Linda Kujanpää, Riikka K. Arffman, Paula Pesonen, Elisa Korhonen, Salla Karjula, Marjo-Riitta Järvelin, Stephen Franks, Juha S. Tapanainen, Laure Morin-Papunen, Terhi T. Piltonen. AOGS; Published Online: June 08, 2022 (DOI: 10.1111/aogs.14382).*

*URL Upon Publication: https://obgyn.onlinelibrary.wiley.com/doi/10.1111/aogs.14382*

*Copyright © 2021 The Cochrane Collaboration. Published by John Wiley & Sons, Ltd., reproduced with permission.*

